# Liraglutide for Lower Limb Perfusion in People With Type 2 Diabetes and Peripheral Artery Disease: The STARDUST Randomized Clinical Trial

**DOI:** 10.1001/jamanetworkopen.2024.1545

**Published:** 2024-03-12

**Authors:** Paola Caruso, Maria Ida Maiorino, Miriam Longo, Chiara Porcellini, Rita Matrone, Lucia Digitale Selvaggio, Maurizio Gicchino, Carla Carbone, Lorenzo Scappaticcio, Giuseppe Bellastella, Dario Giugliano, Katherine Esposito

**Affiliations:** 1 Division of Endocrinology and Metabolic Diseases, University of Campania “Luigi Vanvitelli,” Naples, Italy; 2 Department of Advanced Medical and Surgical Sciences, University of Campania “Luigi Vanvitelli,” Naples, Italy; 3 PhD Program of Translational Medicine, Department of Experimental Medicine, University of Campania “Luigi Vanvitelli,” Naples, Italy

## Abstract

**Importance:**

Peripheral artery disease (PAD) in diabetes may lead to diabetic foot ulcer and lower-extremities amputation. Glucagon-like peptide 1 receptor agonists have proven cardiovascular benefits in trials of people with type 2 diabetes at high cardiovascular risk.

**Objective:**

To examine the effect of liraglutide on peripheral perfusion measured as peripheral transcutaneous oxygen pressure (TcPo_2_) in individuals with type 2 diabetes and PAD.

**Design, Setting, and Participants:**

This open-label randomized clinical trial was conducted between February 1, 2021, and June 30, 2022, with a final follow-up on December 30, 2022, at University of Campania “Luigi Vanvitelli,” Naples, Italy. Fifty-five individuals with type 2 diabetes, PAD, and TcPo_2_ between 30 and 49 mm Hg were included.

**Interventions:**

Patients were randomized to receive 1.8 mg of subcutaneous liraglutide or conventional treatment of cardiovascular risk factors (control group) for 6 months.

**Main Outcomes and Measures:**

Coprimary outcomes were the change from baseline of peripheral perfusion between groups and the comparison of the proportion of individuals who reached 10% increase of TcPo_2_ from baseline in each group.

**Results:**

Fifty-five participants (mean [SD] age, 67.5 [8.5] years; 43 [78%] male) were randomized (27 to the liraglutide group and 28 to the control group) and analyzed. Participants had a median (IQR) hemoglobin A_1c_ level of 6.9% (6.5%-7.8%) and a mean (SD) TcPo_2_ of 40.3 (5.7) mm Hg. Transcutaneous Po_2_ increased over time in both groups, with significant differences favoring the liraglutide group after 6 months (estimated treatment difference, 11.2 mm Hg; 95% CI, 8.0-14.5 mm Hg; *P* < .001). The 10% increase of TcPo_2_ occurred in 24 participants (89%) in the liraglutide group and 13 (46%) in the control group (relative risk, 1.91; 95% CI, 1.26-2.90; *P* < .001). Compared with the control group, individuals in the liraglutide group had a significant reduction of C-reactive protein (−0.4 mg/dL; 95% CI, −0.7 to −0.07 mg/dL; *P* = .02), urinary albumin to creatinine ratio (−119.4 mg/g; 95% CI, −195.0 to −43.8 mg/g; *P* = .003), and improvement of 6-minute walking distance (25.1 m; 95% CI, 21.8-28.3 m; *P* < .001).

**Conclusions and Relevance:**

In this randomized clinical trial of people with type 2 diabetes and PAD, liraglutide increased peripheral perfusion detected by TcPo_2_ measurement during 6 months of treatment. These results support the use of liraglutide to prevent the clinical progression of PAD in individuals with type 2 diabetes.

**Trial Registration:**

ClinicalTrials.gov Identifier: NCT04881110

## Key Points

**Question:**

What is the effect of liraglutide on peripheral perfusion in people with type 2 diabetes and peripheral artery disease (PAD)?

**Findings:**

In this open-label randomized clinical trial of 55 individuals with type 2 diabetes and PAD, liraglutide was associated with a significant increase of transcutaneous oxygen pressure (TcPo_2_) compared with conventional treatment of cardiovascular risk factors. An increase of at least 10% of TcPo_2_ from baseline was achieved by 89% of participants treated with liraglutide and 46% of the control group.

**Meaning:**

The administration of liraglutide improved peripheral perfusion in people with type 2 diabetes and PAD, suggesting that it may prevent the clinical progression of PAD.

## Teaser

This randomized clinical trial of individuals with type 2 diabetes and peripheral artery disease examines the effect of daily administered liraglutide on peripheral perfusion.

## Introduction

Diabetes is considered worldwide as a major health issue, with devastating consequences on morbidity, mortality, and quality of life.^1^ Diabetes is responsible for vascular damage and atherosclerosis, with progressive and systemic complications.^2^ Peripheral artery disease (PAD) is a macrovascular complication of diabetes related to an increase of the risk of cardiovascular events.^3^ Moreover, PAD represents a main determinant of the onset and the prognosis of diabetic foot ulcer, as well as a main cause of lower-extremity amputation in people with diabetes.^4,5^ In 2010, PAD prevalence involved more than 200 million people globally, of whom 20% were affected by diabetes.^6^ The occurrence of PAD in individuals with diabetes shows peculiar features; particularly, it appears earlier, with diffuse and distal location of the atherosclerotic lesions. Clinical manifestations of PAD in individuals with diabetes may be extremely different because of the concomitant presence of diabetic neuropathy, which may determine the lack of symptoms or atypical presentation.^7^ Therefore, diagnosis and follow-up may be challenging.

Both PAD prevention and treatment should include the simultaneous management of all cardiovascular risk factors, including dyslipidemia, hypertension, overweight or obesity, smoking, and hyperglycemia.^2^ Observational studies^3,8^ of people with diabetes reported a 26% increased risk of PAD for each 1% increase in hemoglobin A_1c_ (HbA_1c_). Moreover, glucose control has been associated with the reduction of lower-extremity amputation rate.^3^

Among glucose-lowering medications, glucagon-like peptide 1 receptor agonists (GLP-1RAs) are considered the first-line therapy in people with type 2 diabetes based on their metabolic and cardiovascular efficacy. Indeed, cardiovascular outcome trials have proven the significant cardiorenal benefits of GLP-1RAs in individuals with type 2 diabetes and a history of cardiovascular disease, with a reduction in the incidence of major cardiovascular events.^9,10^ However, although PAD is recognized as a determinant of cardiovascular disease history, it has not been evaluated as an end point in most of the trials. In SUSTAIN-6 (Trial to Evaluate Cardiovascular and Other Long-Term Outcomes With Semaglutide in Subjects With Type 2 Diabetes), semaglutide was associated with a significant reduction of the incidence of revascularization (coronary and peripheral), which was included among the secondary outcomes and mostly driven by coronary revascularization.^11^ Of note, vascular disorders without any specific characterization were addressed as adverse events in all previous cardiovascular outcome trials. The EXSCEL (Exenatide Study of Cardiovascular Event Lowering Trial) trial was the first to report PAD based on the existence of a stenosis of lower-extremity artery of 50% or greater or an ankle-brachial index below 0.9.^12^ Moreover, a post hoc analysis from the LEADER (Liraglutide and Cardiovascular Outcomes in Type 2 Diabetes) trial described a significant reduction in lower-extremity amputations (hazard ratio, 0.65; 95% CI, 0.45-0.95; *P* = .03), mostly driven by major amputations rather than minor ones.^13^ This finding has been related to the liraglutide-induced improvement of several PAD risk factors as well as to pleiotropic effects that liraglutide may exert on the vascular system.^10,14^ Given its potential role in peripheral vascular disease, STARDUST (Effects of the GLP-1 Receptor Agonist Liraglutide on Lower Limb Perfusion in People With Type 2 Diabetes and Peripheral Artery Disease: An Open-Label Randomized Clinical Trial) was designed to assess the effect of daily administered liraglutide on peripheral perfusion in a population of individuals with type 2 diabetes and PAD.

## Methods

STARDUST was a single-center, open-label randomized clinical trial conducted at the Division of Endocrinology and Metabolic Diseases, University of Campania “Luigi Vanvitelli,” Naples, Italy. The STARDUST protocol has been created according to the Consolidated Standards of Reporting Trials (CONSORT) reporting guidelines.^15^ The trial was approved by the University of Campania “Luigi Vanvitelli” ethics committee (see the trial protocol in Supplement 1) and registered on ClinicalTrials.gov. Written informed consent was collected from all participants. Enrollment occurred between February 1, 2021, and June 30, 2022; final follow-up was on December 30, 2022.

### Participant Identification and Inclusion and Exclusion Criteria

**Figure 1.**
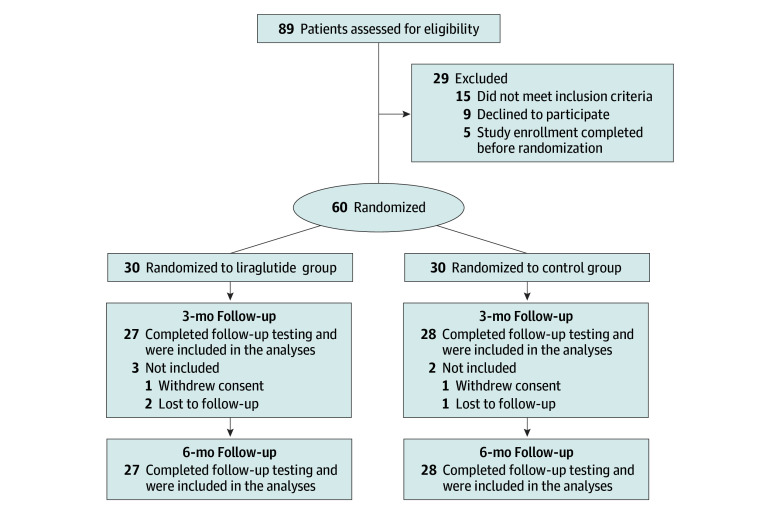
Trial Profile

Between February 1, 2021, and June 30, 2022, a total of 89 patients were screened for eligibility. Participants were recruited from outpatient clinics and/or during hospitalization. Study eligibility was first investigated among individuals 35 years or older with type 2 diabetes and a diagnosis of PAD, evaluated with Doppler ultrasonography, computed tomography angiography, or angiography in the previous 12 months. For recruitment, transcutaneous oxygen pressure (TcPo_2_) of the foot had to range from 49 to 30 mm Hg at the screening visit. Additionally, patients had to have optimal or suboptimal glycemic control (HbA_1c_ ranging from 6.5% to 8% [to convert to proportion of total hemoglobin, multiply by 0.01]) while taking stable doses of glucose-lowering medications. Key exclusion criteria were previous (last 3 months) or current therapy with GLP-1RAs or dipeptidyl peptidase 4 inhibitors, contraindications to the use of GLP-1RAs, current or plans for pregnancy, acute coronary and/or cerebrovascular events within the previous 14 days, plans or indications for peripheral revascularization procedure, estimated glomerular filtration rate below 15 mL/min/1.73 m^2^, neoplasms, psychiatric disorders, and use of drugs or concomitant conditions that precluded the participation in the study. Sixty individuals met the inclusion criteria and underwent randomization: 30 participants were assigned to the liraglutide group and 30 to the control group. Three individuals from the liraglutide group and 2 in the control group withdrew from the study and/or were lost during follow-up; therefore, 55 participants completed follow-up until December 31, 2022, and were included in the final analysis (Figure 1).

### Data Collection

Participants were evaluated at baseline, 3 months, and 6 months to deliver any needed adjustment of the therapies as required by the updated clinical status of the people included. Race, age, sex, smoking habits, and diabetes duration were self-reported. Data on race were collected for each participant in the study as default information on admission. Assessments included cardiovascular events, adverse events, laboratory tests, electrocardiography, vital signs, periodic questionnaires, ankle-brachial index, 6-minute walking test (6MWT), and TcPo_2_ measurements.

### Clinical Evaluation of PAD

Ankle-brachial index evaluation was obtained dividing the leg systolic pressure by the arm systolic pressure, both detected through a Doppler probe and a sphygmomanometer. The 6MWT was recorded for each participant as the maximum possible distance of walking for 6 minutes up and down a 100-m hallway. Transcutaneous Po_2_ evaluation was performed through transcutaneous oximetry (Perimed Inc) at the districts of medial malleolus and the dorsum of the foot, whose perfusion is provided by posterior and anterior tibial arteries, respectively. Patients laid in supine position for 20 minutes. The skin was carefully cleaned with saline solution. The electrochemical transducer was fixed using specific contact liquid and double-sized adhesive rings, avoiding areas overlying bone or superficial vessels. The device was calibrated before each use for at least 5 minutes. The assessment lasted 16 minutes, as recommended by the manufacturer, and was performed in both limbs. The lowest value was used for the analysis.

### Randomization

Participants who met eligibility were randomly assigned to liraglutide subcutaneous daily injection or a matching control group for the duration of the study. Randomization was performed by a computer-generated random number sequence. Allocation was concealed in study folders that were held in a central location until after informed consent was obtained. The medical staff involved in the intervention enrolled participants, assigned them to the trial groups, and conducted the follow-up visits at the hospital, whereas the laboratory staff did not know the patients’ group assignments. The trial staff who assessed outcomes and analyzed the data were masked to group assignment.

### Interventions

Participants randomized to the liraglutide group received 0.6-mg once-daily liraglutide subcutaneous injections at approximately the same time each day. The dose was titrated up on a weekly schedule by a 0.6-mg increase to a target dose of 1.8 mg or the maximum tolerated. Participants who did not tolerate the dose increase were allowed to wait an additional week before attempting the dose titration. Individuals in both groups were given, if needed, tailored therapeutic prescriptions to manage blood glucose levels and cardiovascular risk factors, according to the standards of medical care. The established treatment goals for cardiovascular risk factor control were a systolic blood pressure below 130 mm Hg and diastolic blood pressure below 80 mm Hg obtained, if needed, with angiotensin-converting enzyme inhibitors or angiotensin receptor blockers; a low-density lipoprotein cholesterol level below 70 mg/dL (to convert to millimoles per liter, multiply by 0.0259); and aspirin therapy at a dosage of 100 mg/d or, alternatively, clopidogrel at a dosage of 75 mg/d in case of documented aspirin allergy.^2^

### Outcomes

The coprimary outcomes of the study were the difference in the change from baseline of peripheral perfusion between groups and the comparison of the proportion of individuals who reached a 10% increase of the TcPo_2_ value from the baseline in each group. The TcPo_2_ change was evaluated as the difference between TcPo_2_ values measured at the end of the trial and baseline. Additional secondary outcomes were glycemic and metabolic parameters (HbA_1c_, weight, body mass index [calculated as weight in kilograms divided by height in meters squared], and systolic and diastolic blood pressure), lipid profile (total cholesterol, high-density lipoprotein and low-density lipoprotein cholesterol, and triglycerides), C-reactive protein (CRP), kidney function parameters (urine albumin to creatinine ratio [UACR], and estimated glomerular filtration rate). The STARDUST protocol also included assessment of 6MWT as an exploratory outcome.

### Statistical Analysis

The sample size calculation was performed based on the change of the coprimary end points (change in TcPo_2_). Assuming an estimated SD of 5 mm Hg and a dropout rate of 10%, we expected to require at least 50 participants (in a 1:1 ratio of liraglutide and control strategies) to achieve the 90% power to detect a 10% difference between group at an α = .05.

Continuous variables were described as either means (SDs) or medians (IQRs) according to sample distribution, and count data were reported as numbers (percentages). Comparison among frequencies was made with the χ^2^ test. To evaluate the change of variables over time, we subtracted the values at the start from values at the end. A 2-sample *t* test was used to compare differences between the interventions. The χ^2^ test was used for comparing proportions of participants in the 2 groups who reached the primary end point after the intervention. Relative risks (RRs) and 95% CIs were calculated to demonstrate the relationship between the intervention and the achievement of the primary end point. The analysis of the exploratory outcome (6MWT) included all participants in the study. A 2-sided *P* < .05 was considered statistically significant. All analyses were conducted using SPSS software, version 19.0 (SPSS Inc).

## Results

Fifty-five participants (mean [SD] age, 67.5 [8.5] years; 43 [78%] male and 12 [22%] female; 55 [100%] White) were randomized (27 to the liraglutide group and 28 to the control group) and analyzed. Baseline characteristics of study groups are reported in Table 1 and eTable 1 in Supplement 2. The mean (SD) duration of diabetes was 15.8 (12.8) years, the median (IQR) HbA_1c_ level was 6.9% (6.5%-7.8%), and the mean (SD) TcPo_2_ was 40.3 (5.7) mm Hg. Twenty-one participants received a diagnosis of PAD through Doppler ultrasonography, 15 through computed tomography angiography, and 19 after an angiographic procedure (eTable 2 in Supplement 2). At baseline, 7 participants (13%) reported previous percutaneous transluminal angioplasty of whom 4 individuals (7%) underwent stenting implantation. Five people (9%) reported previous peripheral bypass surgery.

**Table 1.** Baseline Characteristics of the Study Participants

Characteristic	Liraglutide group (n = 27)	Control group (n = 28)
Age, mean (SD), y	67.2 (8.3)	67.4 (8.9)
Sex, No. (%)		
Male	21 (78)	22 (79)
Female	6 (22)	6 (21)
White race	27 (100)	28 (100)
Tobacco use		
Smokers	12 (44)	11 (39)
Not smokers	4 (15)	5 (18)
Ex-smokers	11 (41)	12 (43)
Previous PAD treatment, No. (%)		
PTA	4 (15)	3 (12)
Stenting	2 (8)	2 (8)
Bypass	2 (8)	3 (12)
Diabetes duration, median (IQR), y	11.0 (5.5-25.0)	10.0 (3.0-30.0)
HbA_1c_, mean (SD), %	7.1 (0.5)	6.8 (0.8)
HbA_1c_, mean (SD), mmol/mol	54 (6)	51 (9)
Antidiabetes therapy, No. (%)		
Metformin	18 (75)	20 (77)
Insulin	13 (54)	16 (62)
Other oral drugs	7 (29)	8 (31)
Weight, mean (SD), kg	80.9 (14.3)	81.9 (13.5)
BMI, mean (SD)	29.9 (4.0)	28.1 (4.4)
Blood pressure, median (IQR), mm Hg		
SBP	130.0 (130.0-140.0)	140.0 (130.0-140.0)
DBP	70.0 (70.0-80.0)	80.0 (75.0-80.0)
eGFR, mean (SD), mL/min/1.73 m^2^	73.4 (20.6)	74.1 (15.5)
UACR, median (IQR), mg/g	47.4 (24.1-117.5)	60.0 (39.0-74.3)
CRP, median (IQR), mg/dL	0.4 (0.2-1.5)	0.4 (0.1-1.8)
TcPo_2_, mean (SD), mm Hg	40.0 (5.9)	40.5 (5.7)
ABI, median (IQR)	0.9 (0.9-1.1)	0.9 (0.9-1.1)
6-min walking distance, mean (SD), m	328.3 (14.7)	329.5 (13.3)
Lipids, mean (SD), mg/dL		
Total cholesterol	143.5 (48.6)	155.2 (35.3)
HDL-C	45.9 (14.5)	45.0 (9.7)
LDL-C	76.7 (37.6)	85.6 (28.1)
Triglycerides	104.5 (80.0-133.0)	108.0 (80.0-145.0)
Hypertension therapy, No. (%)		
ACE inhibitors or ARBs	18 (75)	18 (69)
β-Blockers	12 (50)	12 (46)
α-Blockers	2 (8)	2 (8)
Calcium channel blockers	8 (33)	12 (46)
Diuretics	7 (29)	9 (35)
Lipid-lowering therapy, No. (%)		
Statins	18 (75)	20 (77)
Ezetimibe	6 (25)	6 (23)
ω3	4 (17)	4 (15)
Fenofibrate	2 (8)	3 (12)
Antiplatelet therapy, No. (%)	12 (44)	16 (57)
Anticoagulant therapy, No. (%)	12 (44)	16 (57)

Abbreviations: ABI, ankle-brachial index; ACE, angiotensin-converting enzyme; ARB, angiotensin receptor blocker; BMI, body mass index (calculated as weight in kilograms divided by height in meters squared); CRP, C-reactive protein; DBP, diastolic blood pressure; eGFR, estimated glomerular filtration rate; HbA_1c_, glycated hemoglobin; HDL-C, high-density lipoprotein cholesterol; LDL-C, low-density lipoprotein cholesterol; PAD, peripheral artery disease; PTA, percutaneous transluminal angiography; SBP, systolic blood pressure; TcPo_2_, transcutaneous oxygen pressure; UACR, urinary albumin to creatinine ratio.

SI conversion factors: To convert HbA_1c_ to proportion of total hemoglobin, multiply by 0.01; CRP to milligrams per liter, multiply by 10; total cholesterol, HDL-C, and LDL-C to millimoles per liter, multiply by 0.0259; and triglycerides to millimoles per liter, multiply by 0.0113.

### Intervention Adherence

All participants assigned to the liraglutide group tolerated the treatment well for the duration of the study. No down-titration of the dose was required, so all liraglutide group participants took 1.8 mg/d of liraglutide. The numbers of participants in the liraglutide group who underwent changes (dose and/or molecule) of current treatments were 18 (67%) for glucose-lowering, 6 (22%) for lipid-lowering, 6 (22%) for antihypertensive, and 4 (15%) for antiplatelet or anticoagulant medications. In the control group, changes of current therapies occurred in 22 (78%) for glucose-lowering, 9 (32%) for lipid-lowering, 6 (21%) for antihypertensive, and 5 (18%) for antiplatelet or anticoagulant drugs. Mean values of clinical and biochemical parameters assessed at 3 and 6 months for each group are presented in eTable 3 in Supplement 2.

### Coprimary Outcomes

**Figure 2.**
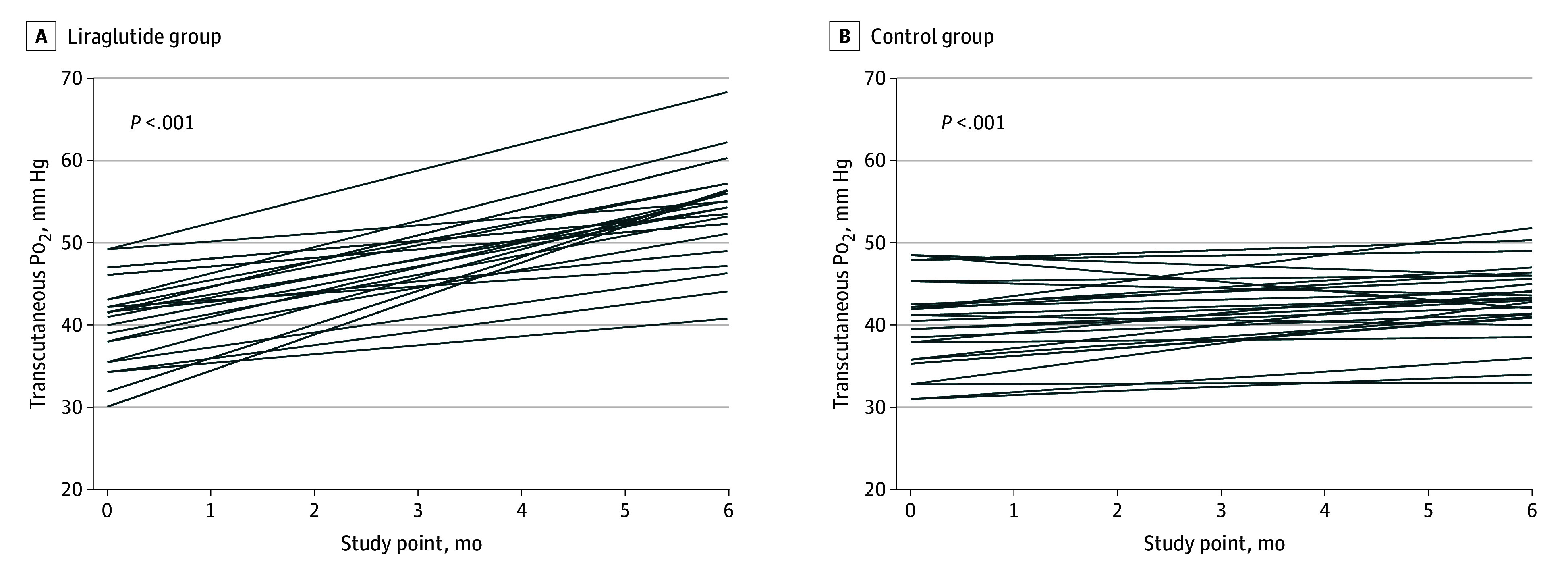
Individual Changes in Transcutaneous Oxygen Pressure

The increase of at least 10% from baseline in TcPo_2_ occurred in 24 participants (89%) randomized to the liraglutide group and 13 participants (46%) assigned to the control group (relative risk, 1.91; 95% CI, 1.26-2.90; *P* < .001). Moreover, TcPo_2_ increased over time in both the liraglutide group (Figure 2A) and control group (Figure 2B), with significant differences favoring the liraglutide group at the end of the trial (estimated treatment difference, 11.2 mm Hg; 95% CI, 8.0-14.5 mm Hg; *P* < .001) (Table 2). This change occurred without differences between groups in glycometabolic profile, including HbA_1c_ levels (eFigure 1 in Supplement 2).

**Table 2.** Comparison of the Changes of Primary and Additional Outcomes Between Groups After 6 Months^a^

Outcome	Δ^b^		Estimated treatment difference for liraglutide vs control group (95% CI)	*P* value
	Liraglutide group	Control group		
Primary outcomes				
TcPo_2_, mm Hg	14.2 (7.1)	2.9 (3.8)	11.2 (8.0 to 14.5)	<.001
No. (%) of patients achieving the primary end point	24 (89)	13 (46)	NA	<.001
Secondary outcomes				
HbA_1c_, %	−0.4 (1.0)	−0.02 (0.4)	−0.4 (−0.8 to 0.01)	.06
Weight, kg	−2.3 (3.2)	−1.2 (5.0)	−1.1 (−3.6 to 1.2)	.33
BMI	−1.1 (2.1)	−0.3 (1.2)	−0.7 (−1.7 to 0.1)	.10
SBP, mm Hg	−5.4 (8.3)	−5.3 (11.8)	−0.03 (−5.8 to 5.8)	.99
DBP, mm Hg	−0.8 (10.0)	−0.3 (10.4)	−0.449 (−6.3 to 5.4)	.87
Total cholesterol, mg/dL	−1.2 (39.6)	3.6 (55.8)	−4.8 (−32.6 to 22.9)	.72
LDL-C, mg/dL	−4.8 (37.5)	8.8 (55.6)	−13.6 (−40.8 to 13.5)	.31
HDL-C, mg/dL	1.2 (8.4)	0.07 (8.6)	1.1 (−3.6 to 6.9)	.63
Triglycerides, mg/dL	−8.0 (49.8)	−7.4 (20.7)	−0.5 (−21.9 to 20.8)	.96
CRP, mg/dL	−0.4 (0.5)	−0.03 (0.6)	−0.4 (−0.7 to −0.07)	.02
UACR, mg/g	−90.5 (161.9)	28.8 (98.5)	−119.4 (−195.0 to −43.8)	.003
eGFR, mL/min/1.73 m^2^	−2.4 (10.9)	0.6 (7.9)	−3.09 (−8.5 to 2.3)	.25
6-min walking distance, m	35.7 (5.8)	10.6 (5.5)	25.1 (21.8 to 28.3)	<.001

Abbreviations: BMI, body mass index (calculated as weight in kilograms divided by height in meters squared); CRP, C-reactive protein; DBP, diastolic blood pressure; eGFR, estimated glomerular filtration rate; HbA_1c_, glycated hemoglobin; SBP, systolic blood pressure; TcPo_2_, transcutaneous oxygen pressure; UACR, urinary albumin to creatinine ratio.

SI conversion factors: To convert HbA_1c_ to proportion of total hemoglobin, multiply by 0.01; CRP to milligrams per liter, multiply by 10; total cholesterol, HDL-C, and LDL-C to millimoles per liter, multiply by 0.0259; and triglycerides to millimoles per liter, multiply by 0.0113.

^a^ Data are presented as mean (SD) unless otherwise indicated.

^b^ Δ refers to the difference between values measured at the end of trial vs baseline within each group.

### Secondary and Exploratory Outcomes

After 6 months, significant differences from baseline for several additional outcomes were observed mostly within the liraglutide group, including HbA_1c_ (mean [SD] change from baseline, −0.4% [1.0%]; *P* = .04), weight (mean [SD] change, −2.3 [3.2] kg; *P* = .002), body mass index (mean [SD] change, −1.1 [2.1]; *P* = .01), systolic blood pressure (mean [SD] change, −5.4 [8.3] mm Hg; *P* = .004), CRP (mean [SD] change, −0.4 [0.5] mg/dL [to convert to milligrams per liter, multiply by 10]; *P* < .001), UACR (mean [SD] change, −90.5 [161.9] mg/g; *P* = .01), and 6MWT measurement (mean [SD] change, 35.7 [5.8] m; *P* < .001). Compared with the control group, individuals assigned to the liraglutide group had a significant reduction in levels of CRP (difference, −0.4 mg/dL; 95% CI, −0.7 to −0.07 mg/dL; *P* = .02) (eFigure 2 in Supplement 2), interleukin 6 (difference, −31.6; 95% CI, −54.6 to −8.7; *P* = .008), luteinizing hormone (difference, −1.1 mIU/mL; 95% CI, −2.0 to −0.2 mIU/mL [to convert to IU/L, multiply by 1]; *P* = .02) (eTable 4 in Supplement 2), UACR (difference, −119.4 mg/g; 95% CI, −195.0 to −43.8 mg/g; *P* = .003) (eFigure 3 in Supplement 2), and 6-minute walking distance (difference, 25.1 m; 95% CI, 21.8-28.3 m; *P* < .001) (eFigure 4 in Supplement 2). Moreover, liraglutide was associated with a higher increase in circulating levels of endothelial progenitor cells and vascular endothelial growth factor A compared with the control strategy at the end of the trial (eTable 4 in Supplement 2). No differences were found between groups in other secondary outcomes (eTables 2 and 4 in Supplement 2). No participants in either group reported adverse events to any medication administered.

## Discussion

This open-label randomized clinical trial of people with type 2 diabetes and PAD showed for the first time, to our knowledge, an increase of peripheral perfusion detected by TcPo_2_ measurement in individuals treated with 1.8 mg of liraglutide compared with people who only managed main cardiovascular risk factors. Moreover, individuals assigned to intervention with liraglutide had a significant reduction of CRP and UACR and a significant improvement of 6-minute walking distance. These results suggest a potential beneficial effect of liraglutide in improving endothelial function in people with type 2 diabetes and PAD.

To the best of our knowledge, randomized clinical trials designed to evaluate the effect of novel glucose-lowering therapies on peripheral perfusion are scanty, although PAD is considered a major cardiovascular event related to morbidity and mortality in diabetes.^16^ In cardiovascular outcome trials designed to demonstrate the cardiovascular safety of novel antidiabetes drugs, a significant reduction of major cardiovascular events was observed in people with type 2 diabetes and history or high risk of cardiovascular disease randomized to liraglutide,^17^ dulaglutide,^18^ albiglutide,^19^ or semaglutide.^11^ Recently, PAD was assessed as an outcome of some of these trials or evaluated in post hoc analyses.^11,12,13^ Most data on the incidence of PAD and its related events in people with diabetes exposed to GLP-1RAs or other glucose-lowering therapies are derived from clinical evidence.^20,21,22,23,24^ These observational studies suggested an improvement of peripheral vascular outcomes in people treated with GLP-1RAs, although they differed in sample sizes, follow-up duration, and estimated end points.^20,21,22,23,24^ Of note, in these studies, heterogeneous definitions of both primary or secondary peripheral vascular outcomes have been provided, so PAD was assessed mostly through the evaluation of its clinical complications or required treatments, including the diagnosis of critical limb ischemia and gangrene or the proportion of hospitalization, percutaneous transluminal angioplasty, or lower-extremity amputation.

STARDUST was specifically designed to prospectively evaluate the potential effect of liraglutide on peripheral perfusion compared with conventional treatment of main cardiovascular risk factors. The primary end point was tested through an objective and reproducible measurement, which is a valuable parameter to detect patients with atypical or absence of symptoms. Indeed, other early diagnostic assessments, including ankle-brachial index, do not offer a reliable threshold to confirm or exclude the diagnosis of PAD. Moreover, the medial wall calcification of lower-extremity arteries, which is common in individuals with diabetes, may interfere with the detection of the blood flow reduction due to atherosclerotic stenosis.^7^ Interestingly, individuals enrolled in both groups did not differ in glycometabolic status at the end of trial or in the management of cardiovascular risk factors provided during the follow-up period. Our findings show that liraglutide improves peripheral perfusion in people with TcPo_2_ values between 30 and 49 mm Hg. This increase was consistent over time within the liraglutide group, with significant differences compared with the individuals randomized to the control group.

Liraglutide, as well as other GLP-1RAs, may determine its effect on peripheral perfusion through both microvascular and macrovascular mechanisms. Liraglutide is effective on glycometabolic outlook, which affects the cardiovascular risk profile.^10,14^ Particularly, GLP-1RAs regulate glucose blood levels and peripheral insulin sensitivity. In addition to the effects on main cardiovascular risk factors, GLP-1RAs may interfere with PAD onset and progression. Previous studies found that exenatide and liraglutide were associated with peripheral vasodilation in both preclinical and observational studies of people with type 2 diabetes.^25,26,27^ Moreover, in the same studies, liraglutide was associated with reduced oxidative stress and intima media thickness, which are both related to endothelial dysfunction.^26,27^ Both preclinical and clinical studies have investigated the relationship among GLP-1RAs, vascular smooth muscle relaxation, and levels of inflammatory markers, including tumor necrosis factor α, plasminogen activator inhibitor 1, interleukin 1β, and interleukin 6, with a favorable effect on chronic systemic inflammation.^28,29^ Furthermore, the activation of the GLP-1 receptor may stimulate endothelial progenitor cells proliferation and differentiation, improving vascular homeostasis, angiogenesis, and plaque stability.^14,30^

Peripheral artery atherosclerosis heavily contributes to lower-extremity complications and hospitalization, with high medical costs and severe clinical burden.^3^ Moreover, PAD and its complications are responsible for the reduction of both life expectancy and quality, with a mortality rate that may overcome cancer mortality.^31^ Particularly, both major and minor amputations, as well as critical limb ischemia, are associated with a 5-year mortality that is inferior only to that related to lung cancer.^31^ According to these data, we could expect that the administration of GLP-1RAs may represent a proper strategy in patients with PAD and diabetes, determining a reduction or a delay of the onset of lower-extremity complications. This finding may be relevant in the prevention of PAD clinical progression because the diagnosis of PAD often occurs with the appearance of critical limb ischemia or gangrene, which usually result in lower-extremity amputation. Moreover, PAD-related amputation in people with diabetes often leads to the permanent disability of the patient with a significant economic burden for the health care system.^3^

### Strengths and Limitations

The main strength is the instrumental assessment of peripheral perfusion through peripheral transcutaneous oximetry. Moreover, enrolled participants were naive to the use of GLP-1RAs and in optimal or suboptimal control of diabetes and other cardiovascular risk factors.

This study also has some limitations. Results were obtained during a 6-month follow-up; therefore, a long-term evaluation should be performed to investigate the durability of the effect. The open-label design could have introduced some biases, although investigators who evaluated the outcomes were blinded to the treatment allocation. The sample size was relatively small, although it was statistically appropriate to evaluate the coprimary outcomes. The study population included a small number of women, although this is consistent with PAD epidemiology.

## Conclusions

This randomized clinical trial found that in people with type 2 diabetes and PAD, the administration of liraglutide significantly increased peripheral perfusion during a 6-month period compared with conventional treatment of cardiovascular risk factors. Whether this effect is associated with other GLP-1RAs should be clarified in future studies.

## Notes

Supplement 1.
Trial Protocol

Supplement 2. **eTable 1.** Additional Baseline Characteristics **eTable 2.** PAD Diagnostic Procedure **eTable 3.** Primary and Secondary Outcomes After 3 and 6 Months in Liraglutide Group and Control Group **eTable 4.** Comparison of the Changes of Secondary Outcomes Between Groups After 6 Months **eFigure 1.** Mean Change in HbA1c Levels Over Time in Liraglutide Group and Control Group **eFigure 2.** Mean Change in C-Reactive Protein Levels Over Time in Liraglutide Group and Control Group **eFigure 3.** Mean Change in UACR Over Time in Liraglutide Group and Control Group **eFigure 4.** Mean Change in 6-Minutes Walking Distance Over Time in Liraglutide Group and Control Group

Supplement 3.
Data Sharing Statement
