# Error in Table and Results

**DOI:** 10.1001/jamanetworkopen.2025.47627

**Published:** 2025-11-12

In the Original Investigation titled “Liraglutide for Lower Limb Perfusion in People With Type 2 Diabetes and Peripheral Artery Disease: The STARDUST Randomized Clinical Trial,”^1^ published March 12, 2024, in Table 2 and the Results, the 6-minute walking distance data erroneously represented the absolute mean values after 6 months, instead of the mean change over 6 months. The correct mean (SD) values are 35.7 (5.8) meters for the liraglutide group and 10.6 (5.5) meters for the control group. This article has been corrected.^1^
