# Supplementary material for: Liraglutide for Lower Limb Perfusion in People With Type 2 Diabetes and Peripheral Artery Disease: The STARDUST Randomized Clinical Trial
Source: JAMA Netw Open. 2024 Mar 12;7(3):e241545. doi: 10.1001/jamanetworkopen.2024.1545 (PMC10933706; doi:10.1001/jamanetworkopen.2024.1545)
Supplement: Supplement 1. — Trial Protocol [file jamanetwopen-e241545-s001.pdf]

EFFECTS OF THE GLP-1 RECEPTOR AGONIST LIRAGLUTIDE ON LOWER LIMB PERFUSION IN PEOPLE WITH TYPE 2 DIABETES AND PERIPHERAL ARTERY DISEASE: A RANDOMIZED CONTROLLED TRIAL (STARDUST)

ClinicalTrials.gov identification number: NCT04881110

Study Protocol (version 2_0401_2021)

Principal investigator: Prof. Katherine Esposito

Full professor of Endocrinology and Metabolic Diseases, University of Campania Luigi Vanvitelli

Chief of the Division of Endocrinology and Metabolic Diseases, University Hospital of Campania Luigi Vanvitelli

Introduction

Diabetes represents a severe social and health burden. Among chronic complications of diabetes, peripheral artery disease (PAD) is involved in diabetic foot ulcer onset and prognosis. PAD diagnosis is based on several assessments: ankle-brachial index (ABI) is a common and simple evaluation. However, it can often be unreliable because of arterial calcification, which is frequent in people with diabetes. Transcutaneous oxygen tension ($TcPO_2$) evaluation is a non-invasive approach to measure tissue's perfusion and oxygenation. Moreover, it is currently recommended to detect the grade of peripheral ischemia in individuals with diabetic foot ulcers. Furthermore, low values of $TcPO_2$ have been associated with the high risk of lower limb amputation, reduction of the healing rate and major cardiovascular events.

Optimal glycemic control has been related to the reduction of the incidence of chronic complications of diabetes. Moreover, among novel glucose-lowering therapies, GLP-1 receptor agonists (GLP-1RAs) have proven over time cardiovascular benefits in cardiovascular outcome trials. Indeed, GLP-1 receptors are widely expressed in endothelial and cardiovascular cells; this may contribute to improve endothelial function and angiogenesis, prevent endothelial cells apoptosis and reduce oxidative stress and inflammation.

GLP-1RAs have determined a significant reduction of major cardiovascular events in cardiovascular outcome trials in people with type 2 diabetes. Moreover, a post-hoc analysis of LEADER trial has described the safety of liraglutide (vs. placebo) in terms of risk of diabetic foot ulcers and/or infection and peripheral revascularization. Of interest, a significant reduction of the incidence of lower extremities amputation occurred in participants of the study with diabetic foot syndrome who were treated with liraglutide.

Aims

“Effects of the GLP-1 receptor agonist liraglutide on lower limb perfusion in people with type 2 diabetes and peripheral artery disease: a randomized controlled trial (STARDUST)” is designed to determine the effect of injectable liraglutide on peripheral perfusion in individuals with type 2 diabetes and PAD. Peripheral perfusion will be assessed through the $TcPO_2$ measurement on the foot. Moreover, we will evaluate the effect of GLP-1RAs on parameters of glyco-metabolic control, inflammation, angiogenesis and cardiovascular risk markers. Particularly, we will analyze:

- glyco-metabolic parameters [fasting glucose, glycated hemoglobin (HbA_{1c}), weight, body mass index (BMI), waist circumference, systolic and diastolic blood pressure];
- lipid profile (total cholesterol, LDL cholesterol, HDL cholesterol, triglycerides);
- inflammation markers [C-reactive protein (CRP), fibrinogen, tumor necrosis factor alpha, interleukin-6];
- renal function parameters [creatinine, azotemia, urine albumin to creatinine ratio (UACR), glomerular filtration rate (eGFR)];
- angiogenesis markers [endothelial progenitor cells (EPCs) and vascular endothelial growth factor (VEGF)];
- PAD clinical assessment (ABI, 6-minutes walking test);
- sexual hormones levels and sexual function.

Study design

STARDUST is a single-center, randomized clinical trial lasting 24 weeks. The study will include a screening visit, a randomization visit and 2 follow-up visits (3 month and 6 month).

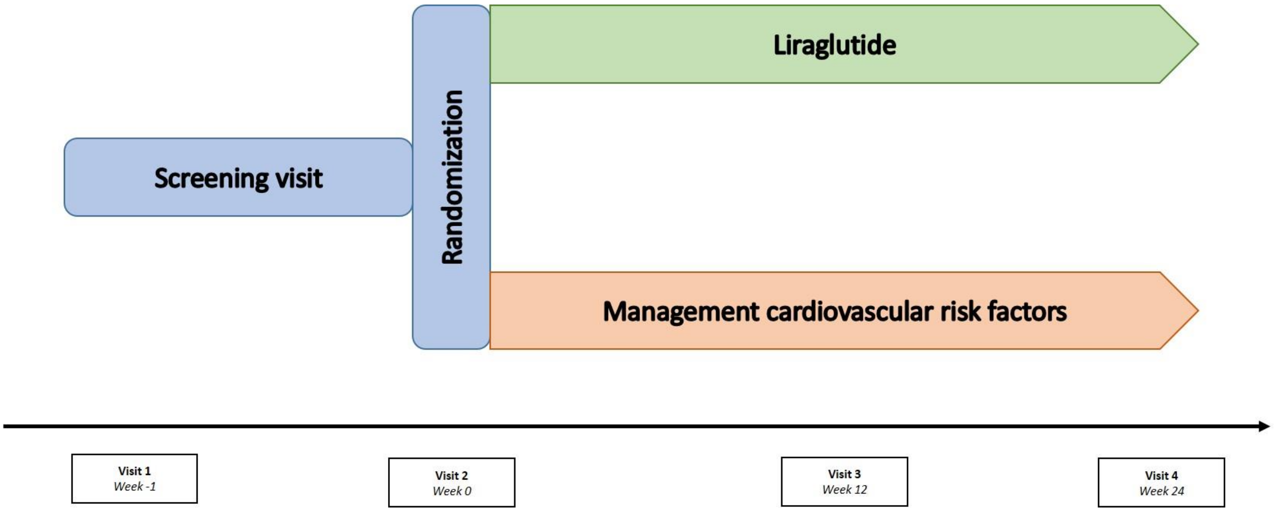

Study population

Eligibility will be considered for individuals with type 2 diabetes aged > 35 years, PAD diagnosed through doppler ultrasound, angio-CT, angiography in the past 12 months, $TcPO_2$ value of the foot ranged between 49 and 30 mmHg.

Inclusion criteria

- diagnosis of type 2 diabetes
- HbA_{1c} ranged between 6.5% and 8%
- Glucose-lowering therapy with metformin and/or insulin on stable doses for at least 3 months

Exclusion criteria

- diagnosis of type 1 diabetes
- current or recent treatment with GLP-1RAs and/or DDP-4 inhibitors
- participation to other clinical research, including drug administration
- contraindications to the treatment with GLP-1RAs
- plans for pregnancy or current pregnancy or breast feeding
- history of thyroid disease
- inflammatory bowel disease
- acute coronary and/or cerebravascular disease within the previous 14 days
- plans and/or indications to peripheral revascularization procedure
- estimated glomerular filtration rate (eGFR) below 15 ml/min per $1.73m^2$
- previous history or current diagnosis of neoplasms and/or anti-neoplastic therapies within 5 years from randomization
- history of diabetic chetoacidosis
- current therapies with steroids and/or anti-psychotic medications
- any concomitant conditions which can preclude participation in the study

Female participants in fertile age have to use contraceptives during the study. Moreover, women who meet inclusion criteria will be screened for pregnancy before the completion of the enrollment.

Randomization

Randomization using a computer generated random-number sequence.

Allocation

Concealed in sealed study folders that will be held in a central, secured location until after informed consent will be obtained.

Interventions

LIRA GROUP**:** participants assigned to LIRA group will start on 0.6 mg once daily liraglutide subcutaneous injection at approximately the same time of the day. The dose will be titrated up on a weekly schedule by 0.6 mg increase to a target dose of 1.8 mg or the maximum tolerated. In accordance with standards of medical care, cardiovascular risk factors will be managed as follows:

systolic blood pressure below 130 mmHg and diastolic blood pressure below 80 mmHg obtained, if needed, with ACE inhibitors or angiotensin receptor blockers, LDL-cholesterol below 70 mg/dL, aspirin therapy at the dosage of 100 mg daily or alternatively clopidogrel 75 mg daily in case of documented aspirin allergy.

CONTROL GROUP*:* individuals assigned to control group will be given, if needed, tailored therapeutic prescriptions to manage blood glucose levels and cardiovascular risk factors, according to the standards of medical care. The established treatment goals for cardiovascular risk factors control will be: systolic blood pressure below 130 mmHg and diastolic blood pressure below 80 mmHg obtained, if needed, with ACE inhibitors or angiotensin receptor blockers, LDL-cholesterol below 70 mg/dL, aspirin therapy at the dosage of 100 mg daily or alternatively clopidogrel 75 mg daily in case of documented aspirin allergy.

Recruitment and time schedule

Recruitment will last 18 months. Participants will be followed up for 24 weeks (6 months).

Time schedule	**Visit 1 (Screening)**<br><br>***Week -1***	**Visit 2 (Randomization)**<br><br>***Week 0***	**Visit 3 (3 months follow-up)**<br><br>***Week 12***	**Visit 4 (End of the study)**<br><br>***Week 24***
Informed consent	x			
Eligibility evaluation	x			
Pregnancy screening test[†]	x			
Start of the intervention		x		
Anamnesis and clinical assessment	x			
Physical examination: weight, BMI, WC		x	x	x
Blood pressure evaluation		x	x	x
Glycemic profile evaluation: fasting glucose, HbA_{1c}	x	x	x	x
Lipid profile evaluation: total cholesterol, LDL-chol, HDL-chol and triglycerides		x	x	x
Renal fuction evaluation: creatinine, azotemia, eGFR	x	x	x	x
Inflammation markers: CRP, fibrinogen, TNF-α and IL-6		x		x
Angiogenesis markers: EPCs and VEGF		x		x
Sexual hormones evaluation: testosterone or estradiol, LH, FSH, SHBG*		x		x
Albuminuria		x		x

ABI and 6-minute walking test		x		x
$TcPO_2$	x	x	x	x
IIEF-5 questionnaire*		x		x
FSFI questionnaire †				
Adverse events evaluation		x	x	x
**In males; †In females.*				

ABI, Ankle Brachial Index; BMI, Body Mass Index; CRP, C-reactive protein; EPC, Endothelial Progenitor Cell; eGFR, estimated Glomerular Filtration Rate; FSFI, Female Sexual Function Index; FSH, Follicle-stimulating hormone; IL-6, Interleukin 6; IIEF-5, International Index of Erectile Function 5; LH, Luteinizing hormone; SHBG, Sex hormone-binding globulin; TcPO2, Transcutaneous Oxygen Pressure; TNF-α, Tumor Necrosis Factor α; VEGF, Vascular Endothelial Growth Factor; WC, Waist circumference.

Study withdrawal

Subjects may withdraw from the research at any time for:

- voluntary retirement;
- occurrence of adverse event which requires the end of the study;
- poor adherence to trial intervention and/or follow-up;
- modification of inclusion/exclusion criteria from baseline;
- novel treatment with medications interfering with the endpoints evaluation;
- pregnancy.

Investigators may interrupt the research in case of:

- occurrence of adverse events (nausea, vomit, abdominal pain, fever, rush and/or eosinophilia);
- renal function worsening (eGFR <15 mL/min/1.73m^2);
- dialysis or renal transplantation;
- chetoacidosis (confirmed by laboratory tests);
- occurrence of acute or chronic pancreatitis;
- medullary carcinoma diagnosis after randomization;
- any adverse event or relevant clinical event, considered as an interference for the research;
- any allergic reactions;
- recruitment of the individual in other study interfering with the research.

Primary outcomes

The co-primary outcomes of the study are the peripheral perfusion assessed at baseline and at the end of the study and the proportion of individuals who reaches a 10% increase of the $TcPO_2$ value from the baseline in each group. The change of $TcPO_2$ of the foot in people treated with liraglutide compared with individuals of the Control group will be calculated after 6 months. The $TcPO_2$ change will be evaluated as the difference between $TcPO_2$ values measured at the end of the trial and baseline.

Secondary outcomes

The secondary outcomes of the study are the change (measured as the difference of variables at the end of the trial vs. baseline) in glyco-metabolic, inflammation and angiogenesis, atherosclerosis

parameters in people treated with liraglutide compared with individuals of the Control group. Moreover, we will evaluate the changes in renal function, sexual hormones levels and sexual function.

Methods

All individuals will be informed about the aims of the study through a specific information letter. Moreover, during the screening visit, we will collect written informed consent and deliver an information letter for the general practitioner of each participant.

Physical examination - Height and weight will be recorded using the Seca 200 scale, with attached altimeter (Seca, Hamburg, Germany). The WC will be measured in orthostatic position, at anterior-superior iliac spine, using a graduated instrument. BMI will be calculated as weight in kg divided by the height in squared meters (kg/m^2). Blood pressure will be measured three times, with patients in sitting position, after at least 15 minutes of rest.

Laboratory assessments – Blood samples will be collected for the evaluation of glycemia, HbA_{1c}, creatinine, serum lipids, according to the attached time-schedule. Calculation of the glomerular filtration rate will be performed using the MDRD formula. Participants will be tested for levels of testosterone, estradiol, LH, FSH and SHBG according to sex. Serum lipids will be determined by enzyme immunoassay. Albuminuria will be measured on 24-hour urine sample and defined as microalbuminuria for urinary albumin excretion values between 30 and 299 mg/24h and macroalbuminuria for values greater than 300 mg/24h.

Inflammation - Serum samples for cytokines, CRP and VEGF will be stored at -80°C until measurements will be performed. Serum concentrations of IL-6, TNF-α and VEGF will be determined in duplicate using highly sensitive immunoenzymatic kits (Quantikine HS, R&D System, Minneapolis, Minn). High sensitivity CRP will be measured by immunonephelometric method.

Determination of circulating levels of EPCs - Each patient will provide a blood sample to determine EPCs count. Cells derived from peripheral blood will be studied for the evaluation of surface antigen expression by flow cytometry. Mononuclear cells will be isolated from peripheral venous blood by density gradient centrifugation and subsequently incubated for 30 min at 4°C in dark field with monoclonal antibodies conjugated to fluorescein isothiocyanate (FITC) for human CD34+ cells (MAb) (Becton Dickinson, Buccinasco, Bologna, Italy), phycoerythrin (PE) conjugated antibodies for human KDR+ cells (MAB) (R&D Systems, Minneapolis, MN, USA), and allophycocyanin (APC) conjugated antibodies for human CD133+ cells (Miltenyi Biotec, Calderara di Reno, Bologna, Italy). After incubation, a quantitative analysis will be performed using BD FACSCalibur and 1,000,000 events will be acquired from each sample. The use of a morphological gate will be employed for the exclusion of granulocytes. Subsequently, CD34+ or CD133+ cells will be identified in the mononuclear cell fraction and these populations will be examined for simultaneous expression of KDR. CD34+CD133+ cells will be identified in the two-dimensional analysis of the dot-plots. The KDR+ mononuclear cell pool will be identified separately. Cells with triple positivity for the considered markers will be searched within the group of CD34+ cells, due to the double expression of KDR and CD133. Data will be processed using the Macintosh CELLquest software (Becton Dickinson). The set-up of the instrument will be optimized daily by analyzing the

expression of lymphocytes derived from peripheral blood labeled with antiCD4Fitc/CD8, Pe/CD3, Pecy5/CD45 Apc 4-, in combination of 4 colors.

Clinical assessment of atherosclerosis – The ABI of both limbs will be calculated through the ratio between the systolic pressure registered at the posterior tibial artery and the systolic pressure recorded at the brachial artery, after at least 15 minutes of rest and in the supine position. The 6-minute walking test will be performed by each participant, walking up and down in a 100 meters hallway. Individuals will receive instructions to cover as much distance as possible in 6 minutes.

Erectile Function Assessment - Male patients will perform a self-assessment test (IIEF-5) of their erectile function and overall satisfaction with sexual life, referring to the previous 6 months. Erectile dysfunction (ED) will be classified based on the total questionnaire score. Any score ≤ 21 will indicate the presence of ED [mild (score 21-17), mild-moderate (score 16 -12), moderate (score 11-8) and severe (score 7-1)]. The IIEF-5 questionnaire will be administered at visit 2 (week 0) and at the end of the study (week 24).

Assessment of Female Sexual Function – Female sexual function will be studied through the FSFI questionnaire which assesses sexual activity over the past 4 weeks and includes 19 questions. The questionnaire will investigate six distinct domains (sexual desire, arousal, lubrication, orgasm, satisfaction and pain), and provide a total score indicative of global sexual function. A total FSFI score < 26.55 will be used to classify the presence of female sexual dysfunction. The FSFI questionnaire will be administered at visit 2 (week 0) and at the end of the study (week 24).

Evaluation of $TcPO_2$ - The measurement of transcutaneous oxygen tension will be performed on both limbs of each participant. Anterior tibial artery perfusion will be assessed near the base of the third finger, while posterior tibial artery perfusion will be evaluated near the lateral malleolus. The patients will be examined in supine position, after 20 minutes of rest. The application of the electrodes has to avoid areas with bony prominences, superficial veins or skin lesions. Measurement sites will be cleaned with an alcoholic solution, the electrodes will be fixed to the skin through an adhesive ring, after the application of a contact solution. Before each measurement, the oximeter will be calibrated for at least 10 minutes. The $TcPO_2$ measurement will be recorded for 16 minutes. According to manufacturer sheet, $TcPO_2$ value ≥50 mmHg is considered as indicator of a normal perfusion, while $TcPO_2$ value <30 mmHg is referred to critical ischemia. The lowest value of each participant will be considered for the follow-up and the analysis.

Planned statistical analysis

The sample size calculation was performed based on the change of the primary endpoint (change in $TcPO_2$). Assuming an estimated standard deviation of 5 mmHg, a dropout rate of 10%, we expect to require at least 50 participants (in a 1:1 ratio LIRA:Control strategies) to achieve the 90% power to detect an 10% difference between group at a α-level of 0.05.

Safety and Adverse Events

Adverse event (AEs) - Any unexpected event occurred in a patient included in a clinical trial after the administration of a pharmacological product. This event is not necessarily related to pharmacological treatment. Therefore, an AE, may be any unpleasant and unwanted clinical sign (for example, an abnormal laboratory finding), symptom, or disease, which is transiently associated

with the use of a drug. According to the guidelines of the Common Terminology Criteria for Adverse Events (https://www.fda.gov/downloads/Guidances/ UCM174090.pdf), AEs will be divided in different grades.

- Grade 1 (mild): lack of symptoms or mild symptoms, with no indication to specific intervention; clinical or diagnostic observation is required.

- Grade 2 (moderate): moderate symptoms requiring local or non-invasive intervention; it may determine a limitation of daily activities.

- Grade 3 (severe or clinically significant, but not immediately life-threatening): requiring hospitalization. It may determine disability and heavily limit daily activities.

- Grade 4 (life-threatening event): indication to urgent intervention.

- Grade 5: death related to AE.

Adverse drug reaction (ADR) - An ADR is defined as a noxious and unintended response to drug administration. It occurs determining pathological changes with doses which are normally used in humans for prophylaxis, diagnosis, or therapy of a disease. The relationship between the drug administration and the adverse event is reasonably possible and, in any case, cannot be excluded.

Serious Adverse Event (SAE) – A SAE is defined as any adverse clinical event that is fatal or life-threatening, which requires hospitalization and leads to a significant disability. It also includes congenital anomalies/birth defects, or any serious medical event that investigator would report.

Hospitalization will be considered as SAE if:

- duration of hospitalization lasts more than 12 hours;

- hospitalization was not previously planned;

- hospitalization is not connected to an AE.

Disability is defined as the significant loss of any ability useful for carrying out normal vital functions.

Unexpected Adverse Reaction or Suspected Unexpected Serious Adverse Reaction (SUSAR) – A SUSAR is a severe and unexpected reaction which is also considered unpredictable, according to drug information (for example, medication data sheet).

Overview of Adverse Event Evaluation

- Type, frequency, severity and severity/intensity of all AEs
- Number of patients discontinuing treatment due to the occurrence of any AE
- Clinically significant changes in ECG, vital signs and laboratory tests

If any AE/SAE occurs, investigator will have the responsibility to report the suspected adverse drug reaction to the Service of Pharmacovigilance, in accordance with current legislation. Moreover,

serious adverse events must be notified to pharmacovigilance manager within 24 hours of the awareness. Furthermore, SUSARs must be notified to the Authorities and Ethical Committee.

Pregnancy

Pregnancy is an exclusion criteria for the study, since GLP-1RAs have no indications in pregnancy. If pregnancy occurs in patients taking liraglutide:

1) treatment with liraglutide will be immediately stopped;

2) pharmacovigilance manager will receive notification (within 24 hours);

3) women will be monitored until the end of the pregnancy.

References

1) Saeedi P, Petersohn I, Salpea P, et al. Global and regional diabetes prevalence estimates for 2019 and projections for 2030 and 2045: Results from the International Diabetes Federation Diabetes Atlas, 9th edition. *Diabetes Res Clin Pract*. 2019;57:107843. doi: 10.1016/j.diabres.2019.107843.
2) Beagley J, Guariguata L, Weil C, et al. Global estimates of undiagnosed diabetes in adults. *Diabetes Res Clin Pract*. 2014;103(2):150-60. doi: 10.1016/j.diabres.2013.11.001.
3) Gerstein HC. Diabetes: Dysglycaemia as a cause of cardiovascular outcomes. *Nat Rev Endocrinol*. 2015;11(9):508-10. doi: 10.1038/nrendo.2015.118.
4) Potier L, Abi Khalil C, Mohammedi K, Roussel R. Use and utility of ankle brachial index in patients with diabetes. *Eur J Vasc Endovasc Surg*. 2011;41(1):110-6. doi: 10.1016/j.ejvs.2010.09.020.
5) Nam SC, Han SH, Lim SH, et al. Factors a ecting the validity of ankle–brachial index in the diagnosis of peripheral arterial obstructive disease. *Angiology*. 2010;61(4):392-6. doi: 10.1177/0003319709348295.
6) Potier L, Halbron M, Bouilloud F, et al. Ankle-to-brachial ratio index underestimates the prevalence of peripheral occlusive disease in diabetic patients at high risk for arterial disease. *Diabetes Care*. 2009;32(4):e44. doi: 10.2337/dc08-2015.
7) Rooke TW, Osmundson PJ. The influence of age, sex, smoking, and diabetes on lower limb transcutaneous oxygen tension in patients with arterial occlusive disease. *Arch Intern Med*. 1990;150(1):129-32.
8) Gazzaruso C, Coppola A, Falcone C, et al. Transcutaneous oxygen tension as a potential predictor of cardiovascular events in type 2 diabetes: comparison with ankle-brachial index. *Diabetes Care*. 2013;36(6):1720-5. doi: 10.2337/dc12-1401.
9) Fagher K, Katzman P, Löndahl M. Transcutaneous oxygen pressure as a predictor for short-term survival in patients with type 2 diabetes and foot ulcers: a comparison with ankle brachial index and toe blood pressure. *Acta Diabetol*. 2018; 55(8):781-788. doi: 10.1007/s00592-018-1145-8.
10) Nauck MA, Meier JJ, Cavender MA, Abd El Aziz M, Drucker DJ. Cardiovascular Actions and Clinical Outcomes With Glucagon-Like Peptide-1 Receptor Agonists and Dipeptidyl Peptidase-4 Inhibitors. *Circulation*. 2017;136(9):849-870. doi: 10.1161/CIRCULATIONAHA.117.028136.
11) Xiao-Yun X, Zhao-Hui M, Ke C, Hong-Hui H, Yan-Hong X. Glucagon-like peptide-1 improves proliferation and differentiation of endothelial progenitor cells via upregulating VEGF generation. *Med Sci Monit*. 2011;17:BR35-41. doi: 10.12659/msm.881383.
12) Lund A, Knop FK, Vilsbøll T. Glucagon-like peptide-1 receptor agonists for the treatment of type 2 diabetes: Differences and similarities. *Eur J Intern Med*. 2014;25(5):407-14. doi: 10.1016/j.ejim.2014.03.005.
13) Eng J, Kleinman WA, Singh L, Singh G, Raufman JP. Isolation and characterization of exendin-4, an exendin-3 analogue, from Heloderma suspectum venom. Further evidence for an exendin receptor on dispersed acini from guinea pig pancreas. *J Biol Chem*. 1992;267(11):7402-5.

14) Marso SP, Daniels GH, Brown-Frandsen K, et al. Liraglutide and Cardiovascular Outcomes in Type 2 Diabetes. *N Engl J Med*. 2016;375(4):311-22. doi: 10.1056/NEJMoa1603827.
15) Marso SP, Bain SC, Consoli A, et al. Semaglutide and Cardiovascular Outcomes in Patients with Type 2 Diabetes. *N Engl J Med*. 2016;376(9):891-2. doi: 10.1056/NEJMc1615712.
16) Hernandez AF, Green JB, Janmohamed S, et al. Albiglutide and cardiovascular outcomes in patients with type 2 diabetes and cardiovascular disease (Harmony Outcomes): a double-blind, randomised placebo-controlled trial. *Lancet*. 2018; 392(10157):1519-1529. doi: 10.1016/S0140-6736(18)32261-X.
17) Gerstein HC, Colhoun HM, Dagenais GR, et al. Dulaglutide and cardiovascular outcomes in type 2 diabetes (REWIND): a double-blind, randomised placebo-controlled trial. *Lancet*. 2019;394(10193):121-130. doi: 10.1016/S0140-6736(19)31149-3.
18) Dhatariya K, Bain SC, Buse JB, et al. The Impact of Liraglutide on Diabetes-Related Foot Ulceration and Associated Complications in Patients With Type 2 Diabetes at High Risk for Cardiovascular Events: Results From the LEADER Trial. *Diabetes Care*. 2018;41(10):2229-2235. doi: 10.2337/dc18-1094.

The trial was approved by the local ethical committee on 13.01.2021. It was conducted between February 2021 and December 2022 at the Division of Endocrinology and Metabolic Diseases of University of Campania “Luigi Vanvitelli”, Naples, Italy, in accordance with the Declaration of Helsinki.
