# Supplementary material for: Liraglutide for Lower Limb Perfusion in People With Type 2 Diabetes and Peripheral Artery Disease: The STARDUST Randomized Clinical Trial
Source: JAMA Netw Open. 2024 Mar 12;7(3):e241545. doi: 10.1001/jamanetworkopen.2024.1545 (PMC10933706; doi:10.1001/jamanetworkopen.2024.1545)
Supplement: Supplement 2. — eTable 1. Additional Baseline Characteristics eTable 2. PAD Diagnostic Procedure eTable 3. Primary and Secondary Outcomes After 3 and 6 Months in Liraglutide Group and Control Group eTable 4. Comparison of the Changes of Secondary Outcomes Between Groups After 6 Months eFigure 1. Mean Change in HbA1c Levels Over Time in Liraglutide Group and Control Group eFigure 2. Mean Change in C-Reactive Protein Levels Over Time in Liraglutide Group and Control Group eFigure 3. Mean Change in UACR Over Time in Liraglutide Group and Control Group eFigure 4. Mean Change in 6-Minutes Walking Distance Over Time in Liraglutide Group and Control Group [file jamanetwopen-e241545-s002.pdf]

Supplementary Online Content

Caruso P, Maiorino MI, Longo M, et al. Liraglutide for lower limb perfusion in people with type 2 diabetes and peripheral artery disease: a randomized clinical trial. *JAMA Netw Open*. 2024;7(3):e241545. doi:10.1001/jamanetworkopen.2024.1545

eTable 1. Additional Baseline Characteristics
eTable 2. PAD Diagnostic Procedure
eTable 3. Primary and Secondary Outcomes After 3 and 6 Months in Liraglutide Group and Control Group
eTable 4. Comparison of the Changes of Secondary Outcomes Between Groups After 6 Months
eFigure 1. Mean Change in HbA1c Levels Over Time in Liraglutide Group and Control Group
eFigure 2. Mean Change in C-Reactive Protein Levels Over Time in Liraglutide Group and Control Group
eFigure 3. Mean Change in UACR Over Time in Liraglutide Group and Control Group
eFigure 4. Mean Change in 6-Minutes Walking Distance Over Time in Liraglutide Group and Control Group

This supplementary material has been provided by the authors to give readers additional information about their work.

© 2024 Caruso P et al. *JAMA Network Open.*

eTable 1. Additional baseline characteristics.

	Liraglutide group (27)	Control group (28)
Waist circumference, cm	110.6 ± 8.6	106.1 ± 10.0
Fasting Glucose, mg/dL	132.0 (103.0, 152.5)	123.0 (111.0, 137.0)
Renal Fuction		
Creatinine, mg/dL	0.9 (0.8, 1.4)	0.9 (0.8, 1.2)
Azotemia, mg/dL	46.5 (28.0, 71.0)	38.0 (30.0, 50.0)
Inflammatory markers		
Fibrinogen, mg/dL	432.0 (329.0, 495.5)	406.0 (306.0, 471.0)
TNF-α (pg/mL)	9.1 (4.1, 88.5)	9.6 (3.8, 79.1)
IL-6 (pg/mL)	44.7 (36.9, 63.9)	43.9 (29.4, 60.5)
Angiogenesis markers		
VEGF-A (pg/mL)	73.1 (47.2, 141.0)	71.0 (59.2, 116.5)
CD34+CD133+KDR+ (n/10^6 cells)	4.0 (1.5, 5.0)	2.0 (1.0, 4.0)
Sexual hormones and function		
LH (UI/L)*	4.8 (4.0, 13.5)	3.9 (3.3, 5.6)
FSH (UI/L)*	8.8 (5.8, 48.4)	8.6 (7.1, 15.8)
Testosterone (ng/dL)*	326.2 (111.1, 26.2)	389.0 (152.4, 34.1)
SHBG (nmol/L)*	48.2 (30.9, 57.9)	45.9 (43.9, 52.9)
IIEF-5 score*	10.4 (6.2)	12.2 (4.9)

FSH, follicle-stimulating hormone; IIEF-5, International Index of Erectile Function-5; IL-6, interleukin 6; LH, luteinizing hormone; SHBG, sex hormone binding globulin; TNF-α, tumor necrosis factor α; VEGF-A, vascular endothelial growth factor A.

*Evaluated only in men included in the study

eTable 2. PAD diagnostic procedure.

	Liraglutide group (27)	Control group (28)
Diagnostic procedure, n (%)		
Doppler ultrasound	10 (37)	11 (39)
Angio-CT	8 (30)	7 (25)
Angiography	9 (33)	10 (36)

© 2024 Caruso P et al. *JAMA Network Open.*

eTable 3. Primary and secondary outcomes after 3 and 6 months in LIRA group and Control group.

	3 months follow-up		*6 months follow-up*	
	**Liraglutide group (27)**	**Control group (28)**	**Liraglutide group (27)**	**Control group (28)**
$TcPO_2$, mmHg	49.4 (5.7)	42.8 (6.1)	54.2 (5.9)	43.4 (4.7)
HbA_{1c}, %	6.9 (0.5)	7.1 (1.0)	6.7 (0.7)	6.8 (0.8)
Fasting Glucose, mg/dL	116.0 (96.5, 143.0)	106.0 (90.0, 139.0)	118.5 (91.0, 132.5)	117.0 (98.0, 122.0)
Weight, kg	80.7 (15.3)	77.3 (13.9)	78.5 (14.4)	80.7 (12.5)
BMI, kg/m^2	29.3 (3.7)	27.2 (4.3)	28.7 (4.0)	27.7 (4.4)
Waist circumference, cm	112.1 (14.4)	99.9 (13.7)	108.9 (10.3)	104.1 (11.6)
Blood Pressure, mmHg				
SBP	130.0 (130.0, 140.0)	140.0 (120.0, 145.0)	130.0 (120.0, 130.0)	130.0 (125.0, 140.0)
DBP	75.0 (70.0, 82.5)	70.0 (70.0, 80.0)	75.0 (70.0, 80.0)	80.0 (70.0, 80.0)
Lipids, mg/dL				
Total cholesterol	136.7 (43.2)	145.8 (45.9)	142.3 (40.6)	158.8 (55.1)
HDL-cholesterol	41.7 (10.9)	42.5 (9.3)	47.1 (17.6)	45.1 (9.6)
LDL-cholesterol	67.5 (28.3)	85.8 (36.3)	71.9 (30.8)	94.4 (49.3)
Triglycerides	127.5 (86.0, 139.0)	109.0 (87.0, 112.0)	113.0 (89.5, 142.5)	109.0 (91.0, 126.0)
Renal Fuction				
Creatinine, mg/dL	1.1 (0.7, 1.4)	0.8 (0.7, 1.0)	1.0 (0.8, 1.2)	0.8 (0.8, 1.0)
Azotemia, mg/dL	39.0 (28.5, 65.5)	38.0 (34.0, 55.0)	49.5 (29.5, 76.5)	44.0 (37.0, 51.0)
eGFR, mL/min	65.4 (26.6)	79.3 (21.1)	71.0 (18.4)	74.7 (13.4)

Data are presented as mean (SD) or median (IQR). BMI, body mass index; DBP, diastolic blood pressure; eGFR, estimated glomerular filtration rate; HbA_{1c}, glycated haemoglobin; SBP, systolic blood pressure; $TcPO_2$, transcutaneous oxygen pressure.

© 2024 Caruso P et al. *JAMA Network Open.*

eTable 4. Comparison of the changes of secondary outcomes between groups after 6 months.

	Δ Liraglutide group	Δ Control group	Estimated treatment difference for Liraglutide group vs Control group (95% CI)	p value
Secondary outcomes				
Fasting glucose	-14.2 (22.4)	-10.7 (15.5)	-3.4 (-14.3 to 7.4)	0.52
Waist circumference	-1.7 (6.5)	-2.0 (4.6)	0.36 (-2.8 to 3.5)	0.81
Fibrinogen	-49.6 (107.3)	-15.2 (144.2)	-34.4 (-107.2 to 38.3)	0.34
Creatinine	0.1 (0.3)	-0.1 (0.2)	0.1 (-0.04 to 0.3)	0.12
Azotemia	6.4 (15.4)	4.0 (12.6)	2.3 (-5.6 to 10.3)	0.55
TNF-α	-31.7 (40.4)	-27.2 (40.6)	-4.4 (-26.6 to 17.6)	0.68
IL-6	-48.0 (55.5)	-16.3 (23.0)	-31.6 (-54.6 to -8.7)	0.008
VEGF-A	71.3 (67.4)	-16.1 (26.2)	87.4 (59.9 to 15.0)	<0.001
CD34+CD133+KDR+	4 (3)	2 (3)	2 (0.4 to 3.9)	0.017
ABI	-0.02 (0.1)	0.007 (0.1)	-0.03 (-0.09 to 0.01)	0.18
LH*	-0.2 (1.1)	0.9 (1.5)	-1.1 (-2.0 to -0.2)	0.015
FSH*	0.5 (1.6)	-0.1 (1.4)	0.6 (-0.3to 1.6)	0.19
Testosterone*	36.1 (145.7)	6.0 (128.8)	30.1 (-60.2 to 120.4)	0.50
SHBG*	3.7 (14.9)	-5.0 (13.5)	8.7 (-0.6 to 18.1)	0.06
IIEF-5 score*	1.5 (2.5)	0.2 (2.3)	1.2 (-0.09 to 2.6)	0.06

Δ refers to the difference between values measured at the end of trial vs. baseline within each group. Outcome values are expressed as mean (SD). ABI, ankle brachial index; FSH, follicle-stimulating hormone; IIEF-5, International Index of Erectile Function-5; IL-6, interleukin 6; LH, luteinizing hormone; SHBG, sex hormone binding globulin; TNF-α, tumor necrosis factor α; VEGF-A, vascular endothelial growth factor A.
*Evaluated only in men included in the study

© 2024 Caruso P et al. *JAMA Network Open.*

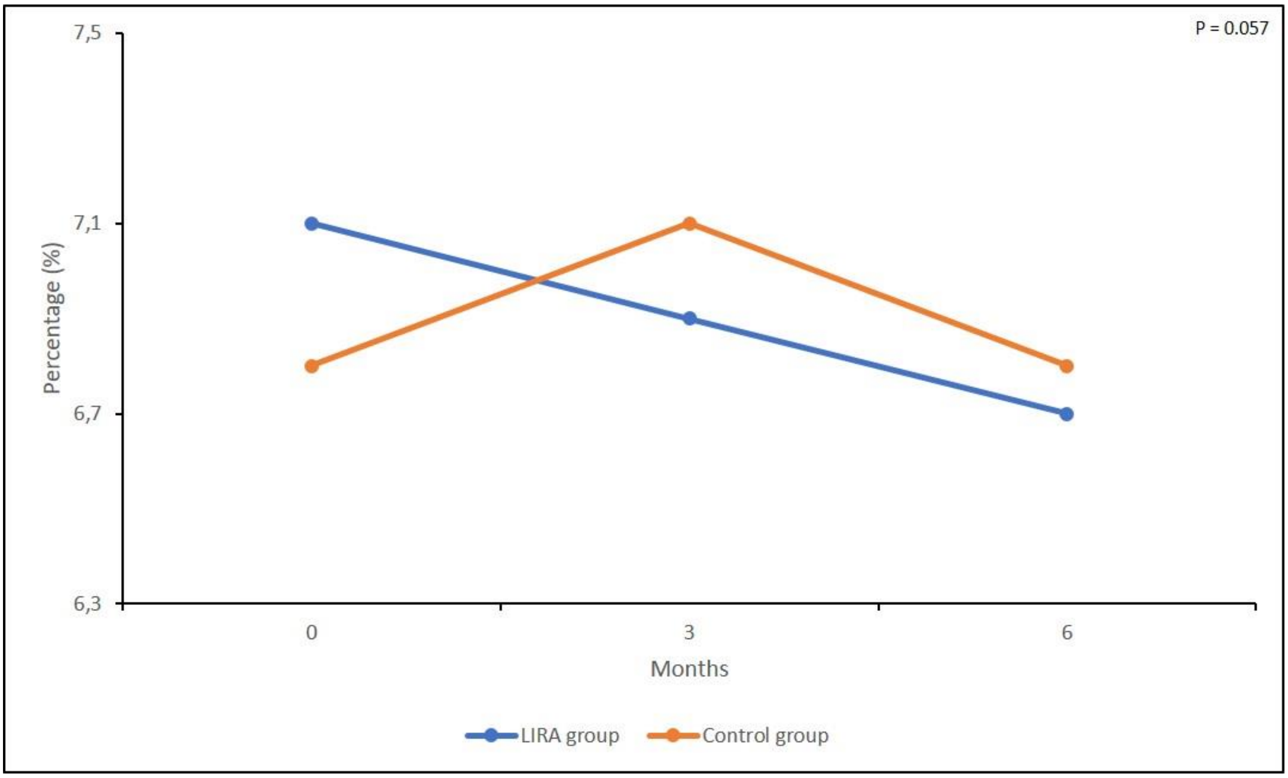

eFigure 1. Mean change in HbA_{1c} levels over time in Liraglutide group and Control group.

© 2024 Caruso P et al. *JAMA Network Open.*

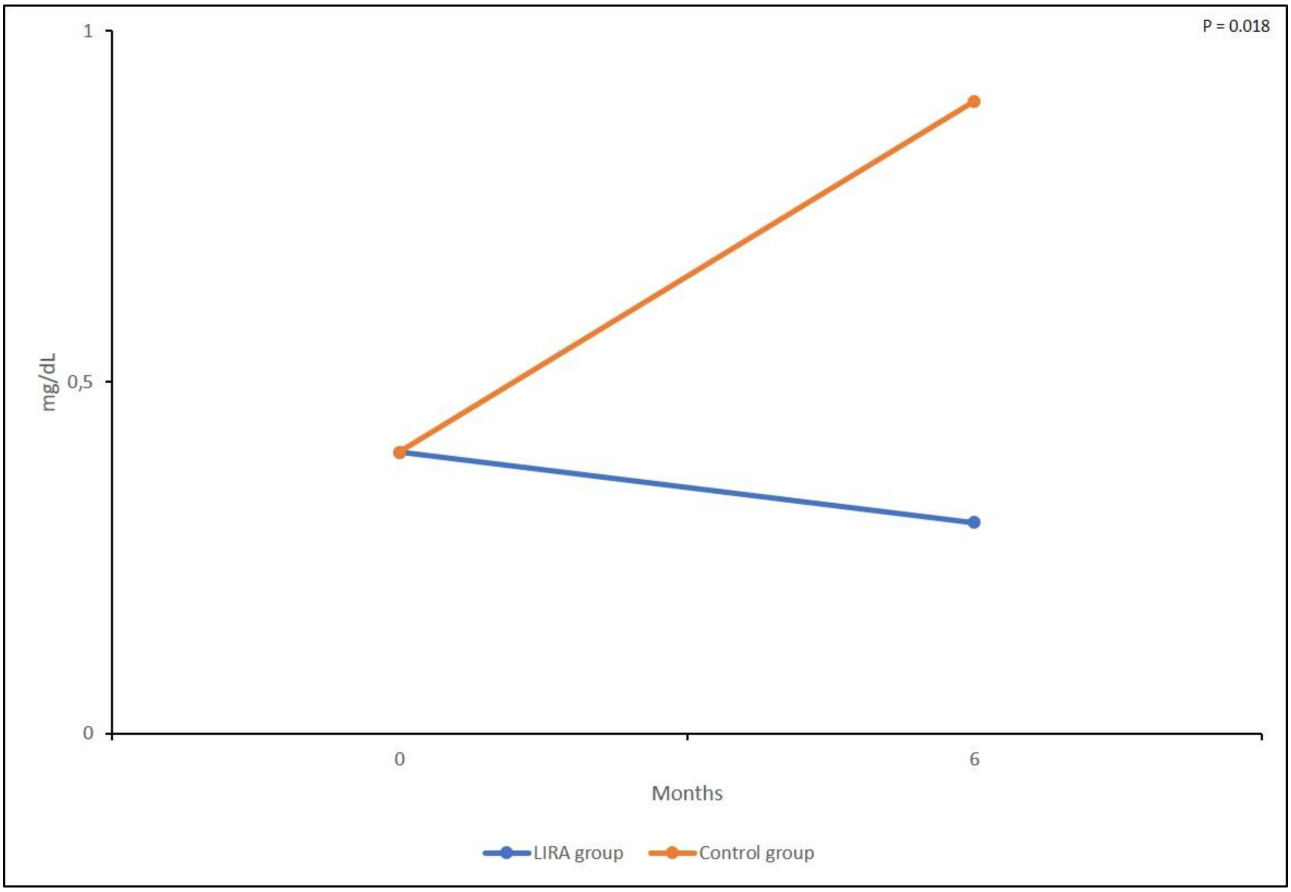

eFigure 2. Mean change in C-reactive protein levels over time in Liraglutide group and Control group.

© 2024 Caruso P et al. *JAMA Network Open.*

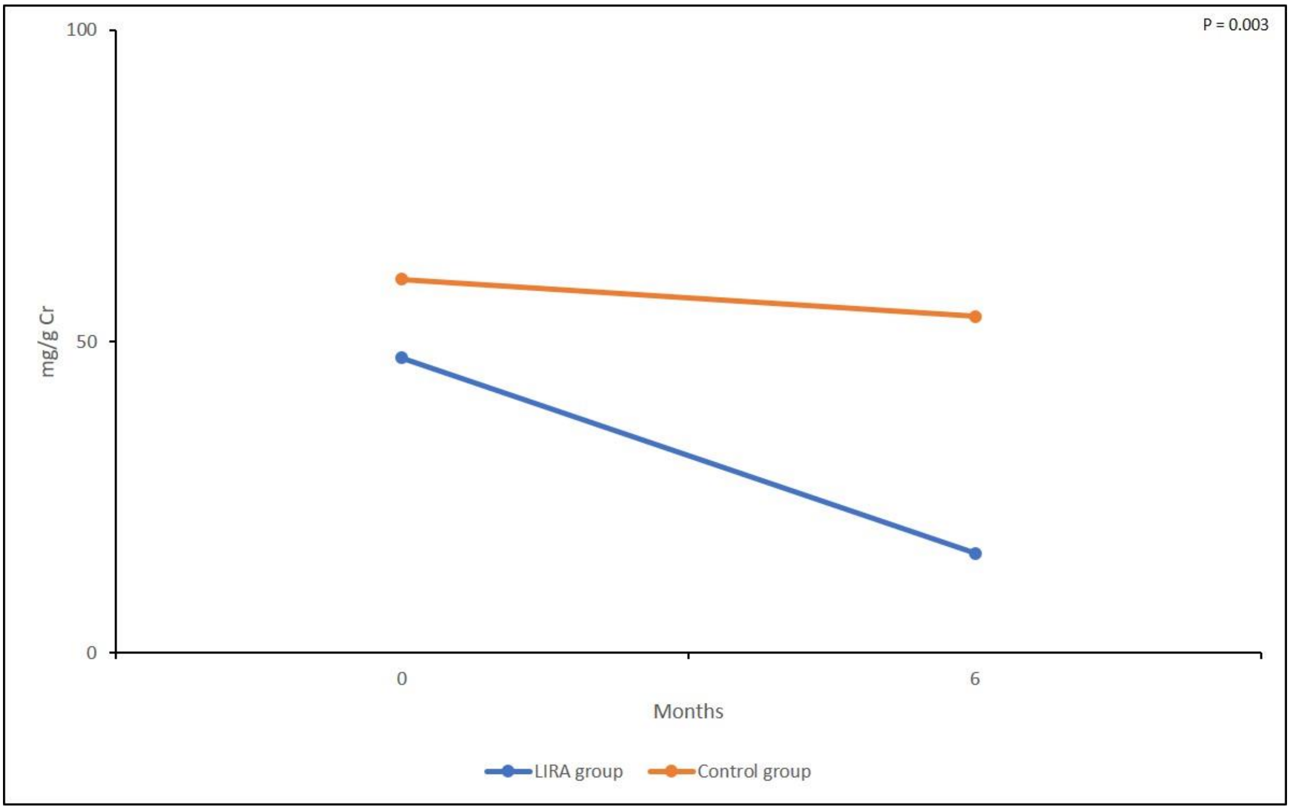

eFigure 3. Mean change in UACR over time in Liraglutide group and Control group.

© 2024 Caruso P et al. *JAMA Network Open.*

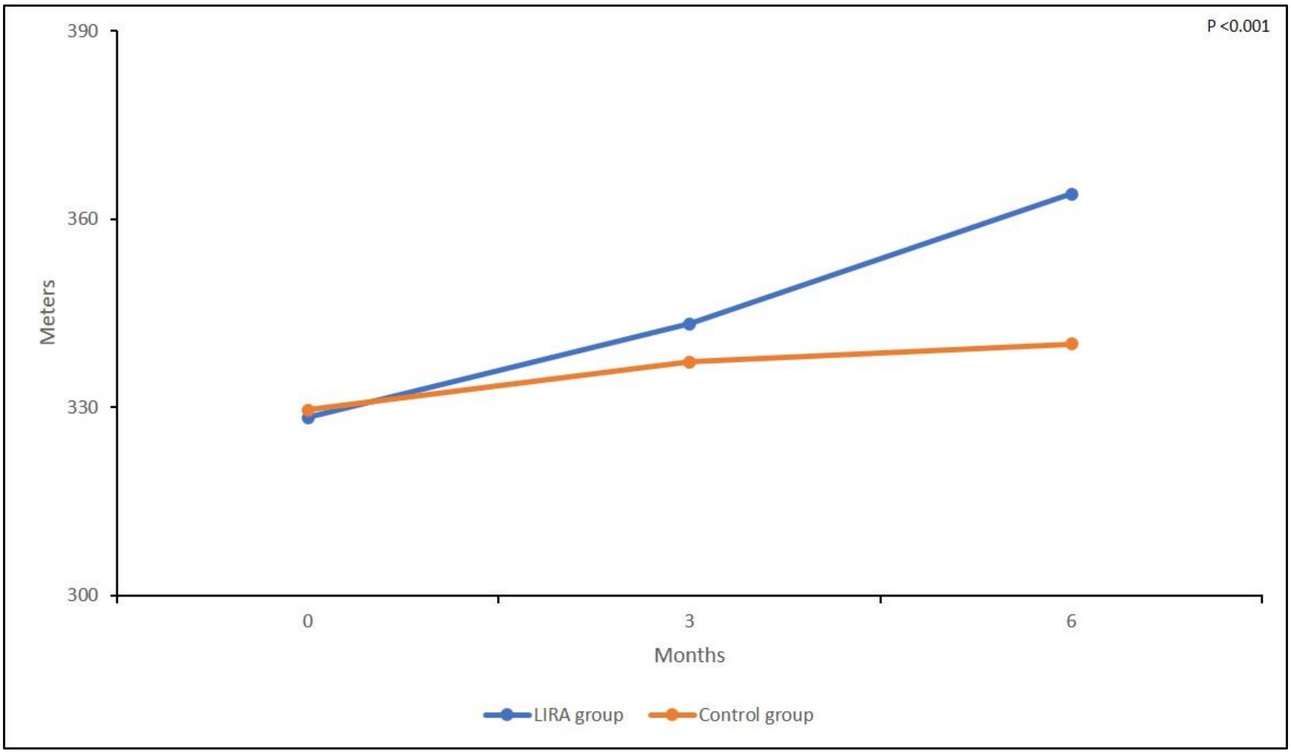

eFigure 4. Mean change in 6-minute walking distance over time in Liraglutide group and Control group.

© 2024 Caruso P et al. *JAMA Network Open.*
