# Supplementary material for: Liraglutide for Lower Limb Perfusion in People With Type 2 Diabetes and Peripheral Artery Disease: The STARDUST Randomized Clinical Trial
Source: JAMA Netw Open. 2024 Mar 12;7(3):e241545. doi: 10.1001/jamanetworkopen.2024.1545 (PMC10933706; doi:10.1001/jamanetworkopen.2024.1545)
Supplement: Supplement 3. — Data Sharing Statement [file jamanetwopen-e241545-s003.pdf]

Data Sharing Statement

Paola Caruso. Liraglutide for Lower Limb Perfusion in People With Type 2 Diabetes and Peripheral Artery Disease. *JAMA Netw Open*. Published March 12, 2024. doi:10.1001/jamanetworkopen.2024.1545

Data
Data available: No

Additional Information
Explanation for why data not available: The data will be provided by the authors on the basis of reasonable requests
